# Complex small supernumerary marker chromosome with a 15q/16p duplication: clinical implications

**DOI:** 10.1186/1755-8166-7-29

**Published:** 2014-04-24

**Authors:** Denise M Christofolini, Flavia B Piazzon, Carolina Evo, Fernanda A Mafra, Stella R Cosenza, Alexandre T Dias, Caio P Barbosa, Bianca Bianco, Leslie D Kulikowski

**Affiliations:** 1 Department of Gynecology and Obstetrics, Genetics Division, Faculdade de Medicina do ABC - FMABC, São Paulo, Brazil; 2 Department of Pathology, Cytogenomics Laboratory, LIM 03, HC-FMUSP, University of São Paulo, Av. Dr. Enéas de Carvalho Aguiar 255, São Paulo 05403-000, Brazil

**Keywords:** Complex sSMC, 15q duplication, 16p duplication, Familial inheritance

## Abstract

**Background:**

Complex small supernumerary marker chromosomes (sSMCs) consist of chromosomal material derived from more than one chromosome and have been implicated in reproductive problems such as recurrent pregnancy loss. They may also be associated with congenital abnormalities in the offspring of carriers. Due to its genomic architecture, chromosome 15 is frequently associated with rearrangements and the formation of sSMCs. Recently, several different CNVs have been described at 16p11.2, suggesting that this region is prone to rearrangements.

**Results:**

We detected the concomitant occurrence of partial trisomy 15q and 16p, due to a complex sSMC, in a 6-year-old girl with clinical phenotypic. The karyotype was analyzed by G and C banding, NOR staining, FISH and SNP array and defined as 47,XX,+der(15)t(15;16)(q13;p13.2)mat. The array assay revealed an unexpected complex sSMC containing material from chromosomes 15 and 16, due to an inherited maternal translocation (passed along over several generations). The patient’s phenotype included microsomia, intellectual disability, speech delay, hearing impairment, dysphagia and other minor alterations.

**Discussion:**

This is the first report on the concomitant occurrence of partial trisomy 15q and 16p. The wide range of phenotypes associated with complex sSMCs represents a challenge for genotype-phenotype correlation studies, accurate clinical assessment of patients and genetic counseling.

## Background

Small supernumerary marker chromosomes (sSMC) are structurally abnormal chromosomes that cannot be identified by banding cytogenetics, and therefore molecular cytogenetic techniques are necessary for their characterization. Part of an sSMC is derived from more than one chromosome. sSMCs have been observed to be derived from translocations [1], and about 64% of complex marker formations are due to parental balanced translocations, while 36% are formed *de novo*. Most of them are of maternal origin (http://ssmc-tl.com/Start.html).

There are balanced translocations in which exchanges of material occur, with no genetic information added or missing, and imbalanced translocations, in which the exchange of chromosome material is unequal, resulting in extra or missing genes [2,3]. The estimated incidence rates of balanced translocation range from about 1 in 500 to 1 in 625 newborns [2]. These translocations are usually harmless, not having any phenotypic effect in most carriers. Later in life, however, they can lead to reproductive problems such as recurrent pregnancy loss, chromosomally imbalanced offspring (including the formation of small chromosome markers), and in some cases infertility, due to the increased risk of generating gametes with unbalanced chromosome translocations [2] and with high levels of DNA fragmentation [4]. Here we report a set of clinical findings from a patient who presents a complex small marker chromosome (sSMC) derived from a maternal translocation between chromosomes 15 and 16, recurrent in her family.

## Case presentation

The patient, BSB, was a 6-year-old girl, the product of the third pregnancy of a healthy, nonconsanguineous young couple with a previous history of two miscarriages. The pregnancy was uneventful. The mother reported that her younger sister also had three miscarriages, and her older sister gave birth to four normal children.

**Figure 1:**
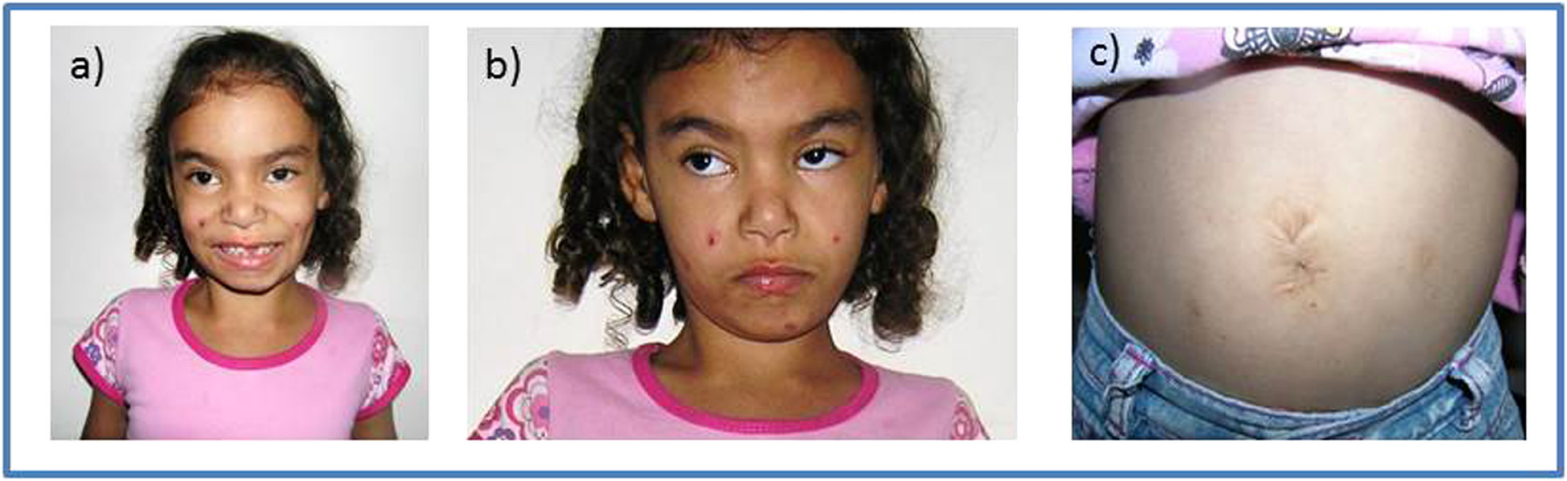
**Propositus at 6 years of age. a)** high-set hairline, mild synophrys, ocular hypertelorism, upslanting palpebral fissures, and wide mouth with conical teeth. **b)** flat-bridged and broad-based nose, hypoplastic nostrils, prominent columella, long filtrum, thin upper lip, prominent chin; **c)** scars of a bilateral inguinal and umbilical hernia correction surgery.

The patient was born by vaginal delivery at 36 weeks of gestation, presenting polyhydramnios, a birth weight of 2750 g (50^th^ percentile) and birth length of 46 cm (50^th^ percentile); her head circumference was 32 cm (50^th^ percentile), and the Apgar scores were 6 and 8. She was kept in intensive care for 3 days due to respiratory distress. At the age of 2 months, she had difficulties in responding to sound stimuli, as a consequence of bilateral otitis. At 6 months, she underwent surgery for the correction of bilateral inguinal and umbilical hernia. Clinical evaluation at the age of six years showed failure to thrive, along with intellectual disability, hearing impairment, speech delay and dysphagia. Physical examination showed a high-set hairline, mild synophrys, ocular hypertelorism, upslanting palpebral fissures, a flat-bridged and broad-based nose, hypoplastic nostrils, prominent columella, long filtrum, thin upper lip, prominent chin and wide mouth with conical teeth. Her hands showed few palmar creases, clinodactyly of the 5^th^ fingers, persistence of digital pads, and her toes had prominent interdigital folds. Some of these features can be seen in Figure 1.

X-rays of hands and feet showed no abnormalities, and a cranial CT scan and MRI were normal. The patient’s early development was severely delayed, with a pronounced deficit in the acquisition of motor, language and social skills. Due to a sucking and swallowing disability, she showed difficulties in gaining weight. She displayed good behavior and a docile personality.

Upon initial analysis, the proposita’s karyotype was determined as being 47,XX,+mar. C-banding and NOR-staining characterized the marker as a monocentric and monosatellited chromosome. A cytogenetic evaluation of the family revealed a 46,XX,t(15;16)(q13;p13.2) karyotype in the mother and one aunt, and a 46,XY,t(15;16)(q13;p13.2) karyotype in the maternal grandfather. The patient’s karyotype was therefore redefined as 47,XX,+der(15)t(15;16)(q13;p13.2)mat.

**Figure 2:**
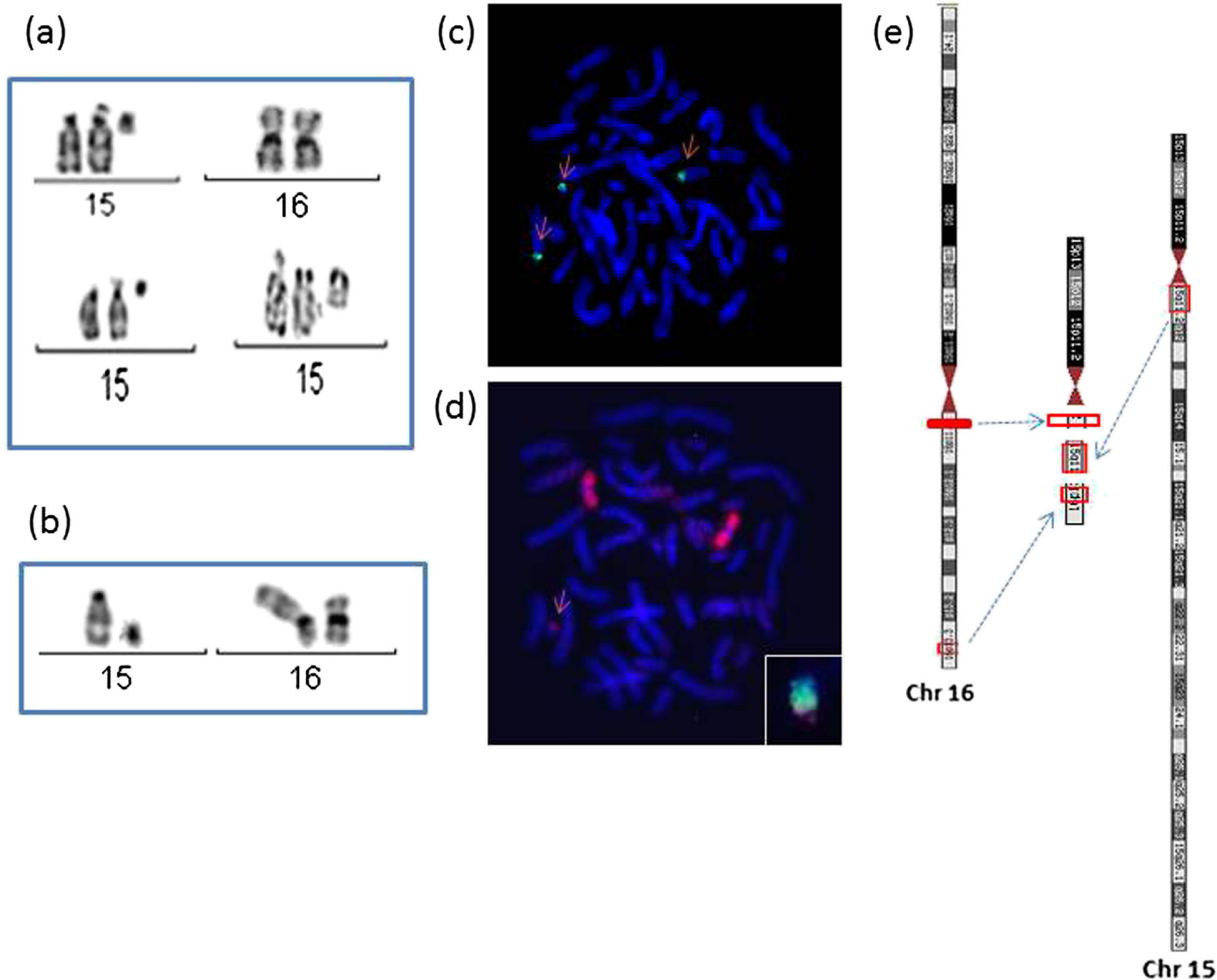
**Cytogenomic results. (a) **Partial karyotype of the patient showing the inherited sSMC using GTG band, C band and NOR staining **(b)** Partial karyotype of the patient´s mother and grandfather **(c; d)** the patient´s sSMC labeled by FISH using a chromosome 15 centromere probe (Aquarius Cytocell) and a chromosome 16 Whole Painting probe (Aquarius® ) and in detail, the sSMC with the co-hybridization showing both probes simultaneously in a single image **(e)** ideogram showing the putative structure of the marker chromosome.

Array-CGH analysis revealed a duplication of about 3.1 Mb of the proximal 15q segment (chr15:18,741,516-21,856,312), comprising *TUBGCP5, CYFIP1, NIPA2 genes*, and unexpectedly also showed a 1.3 Mb duplication of a distal 16p segment (chr16:2,056,890-3,346,212), comprising *TBC1D24; PKD1; THOC6* genes, as well as a small duplication (0.6 Mb) of a proximal 16p segment (chr16:32,748,149-33,316,84). FISH analysis using a centromeric 15 probe and a WCP16 probe confirmed the involvement of chromosomes 15 and 16 in the rearrangement (Figure 2). The breakpoint and intervals of the duplications were entered into the UCSC Genome Browser to search for gene content and function, and were confirmed by the NCBI Map Viewer (http://www.ncbi.nlm.nih.gov/projects/mapview/). DECIPHER (https://decipher.sanger.ac.uk/) and DGV - Database of Genomic Variants: http://dgv.tcag.ca/dgv/app/home were also used to analyze the imbalanced regions.

## Conclusions

In view of these results, the sSMC, initially considered to be a supernumerary der(15), was redefined as a complex marker, derived from the translocated chromosome 15 and containing segments of 15q and 16p.

The presence of sSMCs has been implicated in reproductive problems such as recurrent pregnancy loss, and may be associated with congenital abnormalities in the offspring of carriers. Such abnormalities may arise due to the generation of gametes containing duplicated or deleted chromosome fragments, which may produce individuals with partial trisomies or monosomies [5]. In the present case, we observed a complex marker chromosome formed by a 3:1 segregation, with tertiary trisomy originated from a maternal reciprocal translocation (15;16).

Partial trisomy 15q11-q13 is a well-known neurogenetic disorder that is characterized by clinical heterogeneity. A broad spectrum of moderate to severe symptoms including mental retardation, seizures, poor motor coordination, early-onset central hypotonia, autism spectrum disorders and mild dysmorphic features have been described [6-8]. Our patient had several features in common with previously described cases, including a low nasal bridge, micrognathia, short neck, clinodactyly of fifth fingers, hypotonia, failure to thrive and delayed neuropsychomotor development.

In addition to these, the patient exhibited some unique features, including a high-set hairline, mild synophrys, ocular hypertelorism, upslanting palpebral fissures, flat-bridged and broad-based nose, hypoplastic nostrils, prominent columella, long filtrum, thin upper lip, prominent chin and wide mouth with conical teeth, and hands with prominent interdigital folds.

To our knowledge, there are no other cases in the literature with a duplication of 16p (32.74–33.31 Mb) identical to that presented by our proposita, although the Decipher databases display two reports with similar clinical features. Two other reports describe clinical features associated with duplication of the entire 16p region. In those cases, the phenotype included mental and growth retardation, craniofacial and urogenital abnormalities, abnormal hands and feet, cardiac anomalies, respiratory distress and vascular alterations [9,10].

Tabet et al. [11] discussed the clinical and genetic implications of two different 16p chromosomal rearrangements in a family with three boys affected by autism. Two of the boys were monozygotic twins, displaying – in addition to autism - severe intellectual deficiency, triangular facial structure, deep-set eyes, large and prominent nasal bridge, and a tall-slender build. Both twins presented a *de novo* 16p11.2p12.2 duplication (21.28–30.23 Mb).

Several different duplications and deletions in the 16p 11.2 region (29.5-30.2 Mb) have been described and are associated with dysmorphic features, congenital anomalies and neurobehavioral abnormalities. However, the phenotype of the 16p11 duplication is not well defined.

The proximal 15q chromosome region is highly unstable, as evidenced by its frequent involvement in structural rearrangements. The genomic content of the breakpoints involved in these chromosomal rearrangements offers clues to the potential mechanism behind the instability. Low-copy repeat sequences may be involved in unequal recombination exchanges, due to misalignment during meiosis, leading to chromosomal abnormalities. In addition, the same repeat sequence is located in many places throughout the proximal 15q chromosome region (http://www.ncbi.nlm.nih.gov/pubmed/18177502 - bib18). Five breakpoints were identified within the 15q proximal region and named BP1 to BP5 [12]. The critical region for the Prader-Willi and Angelman Syndromes has been determined to lie between BP2 and BP3 [13]. Here, we observed a 15q breakpoint at BP2.

The short arm of chromosome 16 is also rich in intrachromosomal segmental duplications, which predispose this area to rearrangements. Several recurrent copy number variations involving this region have been recently described. Some of these recurrent rearrangements at 16p11.2 arise through non-allelic homologous recombination (NAHR) between paired segmental duplications [14]. A high incidence of chromosome instability (CIN) was also recently reported in human cleavage-stage embryos, suggesting that germline chromosomal imbalances are subject to reorganization, leading to unexpected complexity [14].

Chromosome shattering followed by re-conjunction of some of the broken pieces (*chromothripsis*) cannot be ruled out as a mechanism occurring in early embryogenesis, possibly involved in the formation of complex marker chromosomes [15,16].

The study provides evidence that the formation of complex sSMCs can have important clinical effects and suggests that in cases of recurrent pregnancy losses, a chromosome investigation should be performed, along with reproductive counseling. Furthermore, we emphasize the importance of using molecular cytogenetic techniques for determining chromosome breakpoints and reaching a better understanding of the mechanisms predisposing certain chromosomal regions to rearrangements.

The wide range of phenotypes associated with sSMCs is a constant challenge for the genotype-phenotype correlation studies, requiring an ever more thorough and detailed clinical assessment of patients and families.

## Methods

### Cytogenetic analysis

Two 5 mL blood samples, collected in heparin and EDTA tubes, were obtained from the proband for cytogenetic and molecular evaluation. One 5 mL blood sample, collected in a heparin tube, was obtained from each one of the parents, one aunt and the grandparents for cytogenetic analysis. Cytogenetic analyses were performed using a standard phytohemagglutinin-stimulated lymphocyte culture method followed by G-banding. Twenty metaphase cells were analyzed for both the patient and her parents. Additional analyses by C-banding and NOR staining were done in the proposita.

### Molecular analysis and array-CGH

Fluorescence *in situ* hybridization (FISH) was performed using a chromosome 15 Alpha satellite probe (Aquarius®, Cytocell, Cambridge, UK) and whole chromosome painting (WCP16) (Aquarius®, Cytocell, Cambridge, UK) according to the technique of Pinkel et al. [17], with minor modifications.

Genomic DNA from the patient and a normal control was isolated from peripheral blood samples using a DNA isolation kit (Promega, Madison, USA). Human genomic DNA from multiple anonymous male donors was obtained from the Promega Corporation (Madison, USA). A microarray assay was performed using the Agilent Human Genome CGH 105A microarray (∼6.4 kb resolution), according to the manufacturer’s protocol version 2.0 for Oligonucleotide Array-Based CGH for high-throughput whole genomic DNA analysis (Agilent Technologies, Inc., Palo Alto, USA).

The array was scanned and analyzed using an Agilent 2565AA DNA microarray scanner (Agilent Technologies, Inc., Palo Alto, USA) and Feature Extraction software. Probes were annotated against the NCBI Build 37 (UCSC hg 18, February 2006). Array CGH results were further validated by FISH.

## Consent

This study was approved by the Research Ethics Committee of Faculdade de Medicina do ABC (Santo André - Brazil). Written informed consent was obtained from the patient’s parents for publication of this Case report and any accompanying images. A copy of the written consent is available for review by the Editor-in-Chief of this journal.

## Abbreviations

sSMC: Small supernumerary marker chromosome; CNVs: Copy Number Variations; FISH: Fluorescence *in situ* hybridization; NAHR: Non-allelic homologous recombination; CIN: Chromosome instability.

## Competing interests

The authors declare that they have no competing interests.

## Authors’ contributions

DMC designed the study and wrote the text, BB participated in the design of the study, FBP performed the clinical evaluation; CE, FAM and SRC performed the classical cytogenetic and FISH analyses; ATD and LDK carried out the cytogenomic analyses; DMC, LDK and CPB coordinated the study and performed a critical analysis of the manuscript. All the authors have seen and approved the final version of the manuscript.
